# Hydroscopic Properties of Organic Objects That May Present as Aural Foreign Bodies

**DOI:** 10.4021/jocmr391w

**Published:** 2010-08-18

**Authors:** Julie C. Brown, Sidrah Rizvi, Eileen J. Klein, Rachel Bittner

**Affiliations:** a University of Washington School of Medicine, Seattle, WA, 98195, USA; b Seattle Children's Hospital, Division of Emergency Medicine, Seattle, WA, 98105, USA; c Seattle Children's Hospital, Clinical and Translational Science, Seattle, WA, 98105, USA

## Abstract

**Background:**

Organic foreign bodies swell when irrigated with water, potentially making extraction more difficult. As the degree and rate of swelling of different types of organic foreign bodies has not been established, we aimed to analyze the hydroscopic properties of different organic foreign bodies in body temperature water.

**Methods:**

Dry kidney beans, brown beans, peas, popcorn kernels, and dried fruits were soaked in a body temperature (37^o^C) water bath. Volume of these organic materials was measured hourly to 8 hours, then at 12, 16, 24, 28, 36 and 48 hours.

**Results:**

All dried fruits and beans increased in volume over time. The volume increase from baseline at 6 hours was between 43% (popcorn kernels) and 383% (kidney beans). Peas, popcorn, and raisins did not increase volume further after 6 hours. Kidney and brown beans had the greatest increase in volume overall (1268% and 482% respectively), and the greatest continued increase after 24 hours.

**Conclusions:**

Many organic substances that frequently present as aural foreign bodies may swell enough in water to lodge tightly in the ear canal. Typical popcorn kernels and dried peas will not swell sufficiently to lodge tightly in the ear canal of a typical child one year or older. A retained organic foreign body in a moist ear canal may cause inflammation until the foreign body can be removed. These risks may be offset by the advantages of successful removal with irrigation.

**Keywords:**

Foreign body; Irrigation; Organic; Ear; Hydroscopic; Procedure; Removal

## Introduction

Approximately 50-80% of children with ear foreign bodies (FBs) have the object removed with simple equipment in the Emergency Department (ED) [1-6]. There are multiple methods of extraction including irrigation, suction, forceps, loops, and hooks. The choice of technique depends on the shape and size of the foreign body, its position in the ear, and provider preference [6-9]. Irrigation is particularly useful for small foreign bodies near the tympanic membrane and for smooth, round objects that are otherwise difficult to grasp [7, 10]. One author recommends starting with the least invasive technique (irrigation) before progressing to more invasive techniques if necessary [11]. While irrigation has been established as an effective mechanism for ear foreign body removal, organic foreign bodies such as beans, seeds, and dried fruit may swell with irrigation, potentially making extraction more difficult. If irrigation attempts fail, organic FB swelling may cause inflammation or make subsequent extraction more difficult [7, 9, 12].

Good data are lacking regarding the impact of irrigation on organic FB size and swelling, and clinical decisions regarding best practice for ear foreign body removal need a stronger empirical base. This is the first study to analyze the hydroscopic properties of potential organic ear FBs in body temperature water, and provides information that will support better clinical practice decisions.

## Methods

We selected organic substances of a size and type typically implicated in ear foreign bodies, including kidney beans, brown beans, dried peas, popcorn kernels, and approximately 4 - 9 mm length or diameter pieces of dried fruit (mangos, apples, raisins, and apricots). We soaked four of each object in a 37^o^C Polytherm water bath (Science/Electronics Inc., Dayton, OH) for 48 hours. Using calipers, we measured the length and width of each foreign body hourly to 8 hours, then at 12 hours, 16 hours, 24 hours, 28 hours, 36 hours, and 48 hours. We estimated volume based on the volume of an ellipsoid (raisins, popcorn kernels, and beans) or a rectangle (cut sections of dried fruit). SAS 9.1.3® statistical software (Cary, NC) was used to determine and graph the mean change in volume and percent change in volume over time for each class of foreign body.

## Results

**Figure 1.**
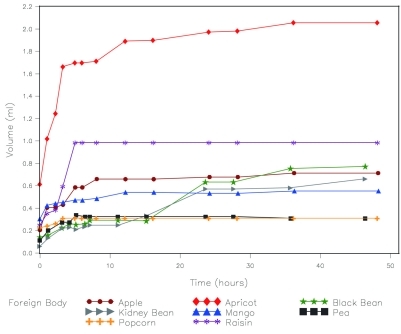
Change in volume over time soaked in water.

**Figure 2.**
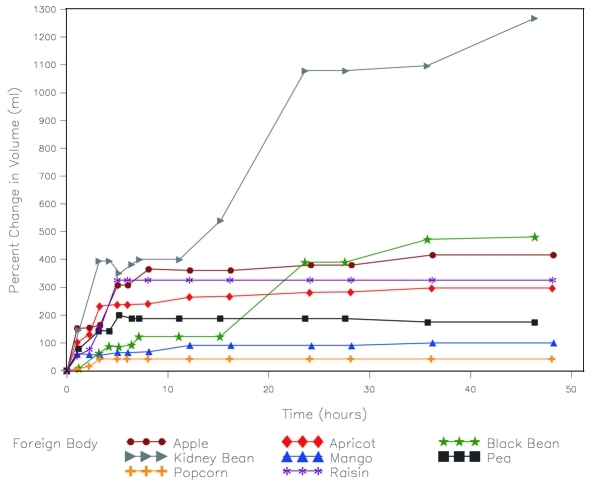
Percent change in volume over time soaked in water.

With the exception of dried mango, the dried fruit gained the most volume over time (Fig. 1). However, this is partly explained by differences in initial size. When percent change in volume is measured, the dried beans and peas had the largest volume change (Fig. 2). Except for kidney beans and black beans, most of the increase in volume took place over the first 12 hours and in some cases did not swell further after 6 hours (Table 1).

**Table 1:** Percent Change in Volume (mls) by Time Point Measured

	3 hours	6 hours	12 hours	24 hours	48 hours
	Mean (SD)	Mean (SD)	Mean (SD)	Mean (SD)	Mean (SD)
Apricot	233 (275.9)	238 (244.3)	265 (279.6)	281 (294.9)	297 (310.1)
Apple	165 (100.1)	307 (224.3)	361 (249.8)	380 (272.8)	416 (313.8)
Black Bean	64 (93.7)	92 (65.6)	123 (84.7)	390 (192.3)	482 (166.7)
Kidney Bean	394 (422.6)	383 (310.6)	400 (297.5)	1080 (786.7)	1268 (925.9)
Mango	56 (42.3)	65 (46.3)	91 (52.2)	90 (43.6)	100 (48.3)
Pea	143 (73.3)	187 (58.0)	187 (58.0)	187 (58.0)	174 (48.1)
Popcorn	43 (30.8)	43 (30.8)	43 (30.8)	43 (30.8)	43 (30.8)
Raisin	151 (59.8)	326 (138.2)	326 (138.2)	326 (138.2)	326 (138.2)

SD = standard deviation

## Discussion

The relative increase in volume over time varied greatly by each organic substance, including the two different types of beans tested. The absolute increase in volume was highly dependent on the initial volume, with small initial volume differences resulting in notable differences in final volume. For many substances tested, the increase in volume with irrigation could be clinically significant relative to the typical ear canal size. The average inner auditory canal diameter is approximately 4.5 mm at its smallest point, and does not change in size after 1 year of age [13]. A spherical object will lodge tightly in an average sized ear canal when the object’s volume is approximately 0.38 ml. The volumes that many common beans and fruit pieces can achieve with hydration exceed the volumes at which they can wedge tightly in an average sized ear canal.

Removal of foreign bodies can be difficult because the ear is pain sensitive and bleeds easily [9]. Successful removal is influenced by the type of foreign body present, its visibility, patient cooperation, and the number of previously failed removal attempts [2, 3, 5, 8]. Although estimates vary widely, approximately 30% of pediatric patients require sedation for successful ear foreign body removal [1, 5, 6, 8, 14]. Hard, regular, smooth objects are among the most difficult to remove [8]. Irrigation, which could otherwise be a useful removal technique, is often not recommended because swelling in a moist ear canal may cause the object to become wedged in a confined space [9, 12]. However, the incidence of failed irrigation attempts and subsequent extraction difficulties may be overstated [1, 11]. In one study that included 17 organic ear foreign bodies, and where 28% of all ear foreign bodies were removed with irrigation, only one object, a coffee bean, required removal by an otolaryngologist [1].

The safety of ear canal irrigation has long been disputed. Oral jet irrigators can generate pressures capable of rupturing the tympanic membrane (TM). In one study, 4 of 5 oral jet irrigators created pressures above the TM rupture threshold, and the fifth had pressures within the range of possible TM rupture [6]. When tested on human cadavers age from 9 months to 91 years, the Sunbeam^TM^ water jet (Elite Electronics Instruments Ltd, Hong Kong) resulted in perforation of 6% of TMs, with 2/3 of the perforations occurring when 1/3 of full power was used. Once the TM is violated, the middle ear and inner ear are vulnerable to damage, including ossicular disruption and perilymph fistula, which can result in severe damage to both hearing and balance [15, 16].

Many techniques and devices may permit safe irrigation in the presence of a normal TM. Cadaveric studies suggest that the pressures generated by a metal ear syringe will not rupture normal TMs but are sufficient to rupture atrophic TMs [17]. Handheld syringing using a 20 ml plastic syringe connected to a 14 gauge cannula produces pressures below the minimum pressure required to perforate an ear drum; subsequent use in 23 children in the ED was successful with no complications [18]. The Propulse^TM^ (Mirage, Bolton, UK), an electronic ear syringe designed for ear wax and foreign body removal, has been shown to successfully remove ear foreign bodies in 88% of 49 pediatric ED cases with no complications [19]. In this study of patients with normal TM integrity, pre-warmed saline (38 - 39^o^C) was directed into the ear along the canal roof, with the head tilted to encourage drainage. The authors claim that the pulsed delivery of fluid prevents pressure buildup, thereby reducing the risk of TM perforation. The OtoClear® Safe Irrigation System (Bionix, Toledo, Ohio) was used for ear wax removal in 18 children (28 ears) with no adverse events, but foreign bodies were not tested [20].

The swelling and softening of organic substances may not always be detrimental. When beans and dried fruit are soaked in water, the softening, disintegration, and splitting that occur may offset increases in volume. We observed that beans and dried fruit often split up, which might make them easier to grasp and may facilitate removal. As evidence to support this theory, we are aware of one case of a child treated in Seattle Children's Hospital ED where softening of a bean appeared to facilitate removal. This 6-year-old child was first evaluated by a primary care provider who applied a topical analgesic solution to the ear canal. After failing to remove the bean, the child was referred to the ED. In the ED, a 6 x 3 mm white bean was found to be tightly impacted in the childs ear canal. The consulted otolaryngologist was able to gently transect the softened bean with a right angle hook, then grasp and remove the two halves. The child remained calm throughout.

In addition to concerns of trauma and swelling with irrigation, there is also a risk of increased ear canal inflammation if a moist organic foreign body is retained for a long time. If irrigation is used and the object is not removed, providers should attempt to gently remove residual water to minimize ongoing swelling and inflammation. The use of an isopropyl alcohol and water solution may result in more rapid evaporation and decreasing subsequent swelling, thereby facilitating further extraction attempts [11].

In conclusion, irrigation may have a valuable place in the removal of select organic foreign bodies, although providers should appreciate the potential for swelling if the object is not quickly removed. Beans, in particular, may continue to swell in a moist environment, for as long as 48 hours. Although organic foreign bodies retained in a moist ear canal may be more likely to cause inflammation until the foreign body can be removed, this risk needs to be weighed against the advantages of successful removal in the ED or clinic. Prompt otolaryngology consultation or follow-up should be arranged for any organic foreign body not removed by ED or clinic providers, particularly if irrigation was used and there is concern for ongoing inflammation and swelling.
